# Neurotoxicity from Old and New Radiation Treatments for Brain Tumors

**DOI:** 10.3390/ijms241310669

**Published:** 2023-06-26

**Authors:** Riccardo Soffietti, Alessia Pellerino, Francesco Bruno, Alessandro Mauro, Roberta Rudà

**Affiliations:** 1 Division of Neuro-Oncology, Department of Neuroscience “Rita Levi Montalcini”, University and City of Health and Science University Hospital, 10126 Turin, Italy; alessia.pellerino@unito.it (A.P.); f.bruno@unito.it (F.B.); roberta.ruda@unito.it (R.R.); 2 Department of Neuroscience “Rita Levi Montalcini”, University of Turin and City of Health and Science University Hospital, 10126 Turin, Italy; alessandro.mauro@unito.it; 3 I.R.C.C.S. Istituto Auxologico Italiano, Division of Neurology and Neuro-Rehabilitation, San Giuseppe Hospital, 28824 Piancavallo, Italy

**Keywords:** cognitive decline, radionecrosis, whole-brain radiotherapy (WBRT), particle therapy, protons, radiosurgery, neuroprotection

## Abstract

Research regarding the mechanisms of brain damage following radiation treatments for brain tumors has increased over the years, thus providing a deeper insight into the pathobiological mechanisms and suggesting new approaches to minimize this damage. This review has discussed the different factors that are known to influence the risk of damage to the brain (mainly cognitive disturbances) from radiation. These include patient and tumor characteristics, the use of whole-brain radiotherapy versus particle therapy (protons, carbon ions), and stereotactic radiotherapy in various modalities. Additionally, biological mechanisms behind neuroprotection have been elucidated.

## 1. Introduction

Improvements in technology have allowed, in recent years, the delivery of more conformal doses of radiation to primary brain tumors in adults, for instance gliomas, thus minimizing the risk of late adverse effects in the normal brain. However, brain metastases or primary central nervous system lymphomas (PCNSL), due to their multiplicity, cannot profit from these conformal advances, and still need treatments covering a major part of the brain. Additionally, in pediatric patients, tumors such as pilocytic astrocytomas, medulloblastomas, or ependymomas are located close to critical normal structures (optic pathways, brain stem), which require as much protection as possible from the damage of radiotherapy.

The aim of this review is to elucidate the biological mechanisms behind the brain damage following different forms of radiotherapy, from the older approaches to the newer ones, and to discuss how biologic advances may lead to new modalities of brain protection.

Research regarding the mechanisms of brain damage following radiation treatments for brain tumors has increased over the years, thus providing a deeper insight into the pathobiological mechanisms and suggesting new approaches to minimize this damage.

## 2. Patient and Tumor Characteristics as Risk Factors for Cognitive Decline

Advanced age is associated with poorer neurocognitive function (NCF) in gliomas [1], while the reverse is true for higher education level, likely reflecting the amount of cognitive reserve.

Some patient genetic characteristics are also associated with NCF. Genes involved in the survival and growth of neurons (BDNF) and neurotransmitter regulation (COMT, DRD2) seem to predict a reduced risk of neurocognitive decline [2,3]. Variants of aging, dopamine, myelin, and DNA repair genes have been associated with NCF in brain tumors [4]. Moreover, patients with gliomas harboring the APOE e4 allele are at increased risk of NCF impairment [5]. 

Tumor location plays a role in the pattern and severity of NCF disturbances. However, diffuse cerebral alterations may be associated with focal lesions as well [6,7]. 

Larger tumor volumes are associated with impaired NCF [8]. Tumor grade, kinetics of growth, and molecular characteristics may influence the risk of brain damage independent of tumor volume: higher-grade gliomas and IDH wild-type tumors are associated with more severe NCF impairment than lower-grade and IDH mutant gliomas, respectively [8,9,10].

## 3. Cognitive Decline Following Whole-Brain Radiation Therapy (WBRT)

### 3.1. Clinical Aspects

Cognitive function is the result of the functional and structural integrity of the brain. The integrity of the hippocampus is critical for memory performances, while the connections between the frontal and parietal cortex and the basal ganglia/thalamus are critical for attention, focus, speed of progression, planning, and executive functions. White matter tracts, which guarantee neural connections, are particularly sensitive to radiation damage [11,12,13].

The cognitive decline following WBRT is well known [14]; it has been reported in up to 30% of patients with brain metastases at 1 year after receiving WBRT alone [15] and in up to 24% of patients with primary central nervous system lymphoma (PCNSL) at 5 years after receiving consolidation WBRT following induction chemotherapy with methotrexate-based regimens [16]. The clinical symptoms of neurotoxicity range from mild short-term memory difficulties to more serious deficits, such as gait disturbances, urinary incontinence, and frank dementia. Older patients (>60–65 years) are more vulnerable to the neurotoxic effects of WBRT. Commonly, long-term survivors develop cognitive defects over time, which is associated with changes on MRI, such as cortical atrophy, hyperintensity of the white matter in T2-FLAIR images, and hydrocephalus. 

Recent clinical trials have also reported an early cognitive decline (at 3 or 4 months) following WBRT, consistent with a significant decline in learning and memory functions and verbal fluency [17,18]. However, it is still unknown whether this early decline is associated with long-term and/or permanent decline (Table 1).

### 3.2. Pathophysiology

In the developing brain, neurogenesis occurs in two critical regions: the subgranular zone (SGZ) of the hippocampus and the subventricular zone (SVZ) of the lateral ventricles. From these regions, multipotent neural stem cells are able to differentiate into neurons and glial cells.

Reduced neurogenesis after exposure of neural precursors (neural stem cells) to radiation either in single or fractionated doses is one of the main mechanisms thought to be responsible for cognitive deterioration, especially memory impairment [19,20,21]. The basic mechanism of the radiation-induced depletion of neural progenitors is an impairment of the mitotic capabilities occurring over the course of multiple cell divisions. After an early reduction, neurogenesis in the SVZ has a delayed recovery, while that in the SGZ remains stalled [22]. 

Irradiation has an effect on neural precursor differentiation, which is influenced by the surrounding microenvironment [19]: in vitro models have shown an increase in differentiation toward both neurons and glia through cell cycle arrest, while in vivo, preferential differentiation along the astrocytic lineage occurs [23].

Neuroinflammation is one of the major changes in the microenvironment following irradiation: the oxidative stress within the brain through the generation of free radicals ultimately results in an upregulation of pro-inflammatory pathways. Due a relative lack of endogenous antioxidants, the normal brain is particularly susceptible to damage. Cranial radiation induces an increase in tumor necrosis factor-α, nuclear factor-KB signaling, and interleukin-6, while reducing brain-derived neurotropic factors that support neuronal development and function [24]. The activation of microglia (the resident immune cells in the brain) is one of the mechanisms behind the increase in neuroinflammation and contributes to the inhibition of neurogenesis with relative sparing of gliogenesis. An interesting finding is that the level and outcome of inflammation vary across different brain structures: for instance, the inflammatory response seems to be persistent after initial irradiation in the hippocampus [25].

In the normal brain, proliferating neural precursor cells are recruited in parallel with the stimulation of angiogenesis, with the cells clustered around small vessels. In the irradiated hippocampus of rats, the relationship between the microvasculature and neural precursor cells is disrupted for several months [19]; these changes could contribute to the prolonged reduction in neurogenesis. 

Overall, very little neurogenesis occurs in the adult brain: thus, deficit in neurogenesis alone may not fully explain the negative effects of radiation on cognitive functions.

Following therapeutic doses of radiation, mature neurons still survive without any change in total number or volume [26], but changes in their ability to transmit information occur. In this regard, both acute and long-term alterations in dendritic morphology and physiological functions of irradiated hippocampal neurons in animals have been reported. Parihar et al. (2013) [27] observed a significant and persistent reduction of dendritic branching, length, and area in a dose-dependent manner. Dendritic spines are small, actin-rich protrusions, which home most excitatory synapsis in the CNS: thus, changes in dendritic spine structure and density correlate with changes in synaptic number and strength. A significant reduction in number (up to 35%) and density (up to 70%) of dendritic spines (in particular of the immature filopodia) has been observed in the hippocampal neurons after irradiation [27,28]. Likewise, Duman et al. (2013) [29] reported that after the exposure of hippocampal neurons to radiation, an acute proliferation of dendritic spines followed by a progressive and persistent loss occurred, reflecting an early increase followed by a late decrease in synapses. Ultimately, alterations in dendritic spines reflect disturbances in synaptic function and plasticity, and thus in neuronal connectivity. Initial studies on hippocampal slices of animals following exposure to radiation showed significant acute changes in the synaptic efficiency in a dose-dependent manner [30,31]. Moreover, a reduction in long-term potentiation (LTP), which describes the strengthening of synaptic connections following high-frequency stimulation, has been demonstrated in the rat hippocampus after radiation [32]. The long-term deficit in synaptic plasticity could reflect the negative effect of radiation on glutamate receptor expression and functioning, including abnormalities in glutamate uptake by both neurons and astrocytes [33,34].

In addition to damage to neural precursors and mature neurons, radiation may disrupt the microvasculature by damaging the blood–brain barrier (BBB), and lead to ischemia and neurotoxicity. It has been well-known for many years [35] that radiation induces mitotic death in endothelial cells with subsequent platelet adhesion to the exposed matrix, which causes thrombus formation and the occlusion of small vessels. Moreover, radiation accelerates the atherosclerotic process by thickening the basement membrane and increasing collagen, thus resulting in vascular insufficiency. Ischemia induces a rise in extracellular glutamate, which will trigger neuronal excitotoxicity through the persistent activation of NMDA receptors [36].

Finally, radiation-induced senescence may occur in astrocytes [37].

Overall, the experimental models (most in rodents) are hardly comparable, as the doses and regimens of radiotherapy were heterogeneous and, last but not least, the translation to human scenarios is hampered by the fact that the effects on the brain seen in animals are acute or subacute, and no data are available regarding late effects.

### 3.3. Strategies to Minimize Cognitive Decline Following WBRT (“Gentler WBRT”) 

Radiation-induced vascular damage is similar to small vessel disease of vascular dementia. Due to the overlapping mechanisms responsible for neurotoxicity in both radiation-induced vasculopathy and vascular dementia, it has been proposed to investigate a compound, such as memantine, with efficacy in vascular dementia, as a protective drug to minimize cognitive deterioration following WBRT. Memantine is a low-affinity voltage-dependent noncompetitive antagonist of NMDA receptors that binds to NMDA receptors and prevents the binding of glutamate when released at high levels in the ischemic milieu. The physiological function of NMDA receptors at the synaptic level is relatively preserved due to the low affinity, noncompetitive nature, and rapid off-rate kinetics of memantine. Memantine gained FDA approval for the treatment of vascular dementia based on two phase III, randomized, placebo-controlled trials [38,39]. The Radiation Therapy Oncology Group (RTOG) 0614 phase II trial investigated the role of memantine in the preservation of cognitive function in patients with brain metastases receiving WBRT through a randomization of WBRT with placebo versus WBRT plus memantine up to 24 weeks [40]. Final results reported in the memantine arm at 24 weeks show a trend toward an improvement in memory function as measured by the Hopkins Verbal Learning Test-Revised Delayed Recall (HVLT-R DR) with significantly less decline in several cognitive secondary endpoints. Moreover, memantine was associated with reduced cognitive function failure.

A second approach to minimize the cognitive decline following WBRT consists in the hippocampal avoidance during WBRT [41]. The rationale of this approach is twofold. First, the hippocampus has a central role in learning and memory functions, especially in the episodic memory (process of forming new memories of events or facts), and an impairment of memory functions with hippocampal atrophy on neuroimaging has been reported in patients receiving radiotherapy for nasopharyngeal, maxillary, pituitary, and skull base tumors [11,42]. Second, neuronal stem cells in the SGZ of the hippocampus, which are responsible for maintaining neurogenesis and preserving memory functions, are extremely sensitive to radiation even at low doses [43]. Based on the fact that the involvement of the hippocampus in the metastatic process is rare, RTOG decided to investigate whether avoiding the irradiation of hippocampus via intensity-modulated conformal radiotherapy techniques (HA-WBRT) could lead to better preservation of memory functions. Preliminary results from a single-arm phase II study were promising, with only 4.5% of patients experiencing tumor progression within the hippocampal avoidance region [44]. 

The phase III trial NRG Oncology CC001 randomized patients with brain metastases to either WBRT + memantine or HA-WBRT + memantine [45]. Time to cognitive deterioration was significantly longer in the HA-WBRT memantine arm, and patient-reported symptoms were improved, while OS and PFS were similar between the two arms. The conclusion was that HA-WBRT + memantine may be considered the standard of care for patients with brain metastases and good performance status who plan to receive WBRT. This statement has been accepted in US guidelines, while in Europe most centers use HA-WBRT without the addition of memantine. A limitation of the two aforementioned trials is that the evaluation of neurocognitive function was performed for a maximum of 6 months; thus, the long-term protective effect is unknown. 

Donepezil is a drug that reversibly inhibits acetylcholine esterase, thus enhancing the level of acetylcholine and the cholinergic transmission in the brain. Donepezil has been shown to increase cerebral perfusion in brain regions critical to cognitive processing in moderate to severe Alzheimer disease [46]. Thus, donepezil has been investigated in a double-blinded, placebo-controlled phase III trial in patients with either primary or metastatic brain tumors to prevent cognitive deficit [47]. The trial failed to demonstrate a difference at 24 weeks in the overall cognitive function between the two arms; however, a modest improvement in several cognitive items in patients with greater pre-treatment deficits was observed.

In order to mitigate the cognitive sequelae in long-term survivors with PCNSL a reduced dose of WBRT has been investigated [48,49,50] with promising results in terms of balance between maintenance of the cognitive integrity and risk of recurrence. However, Correa et al. (2019) [51] have reported delayed cognitive decline (especially in memory functions), occurring between 3 and 5 years following an initial improvement. Interestingly, similarly delayed neurotoxicity was observed in patients receiving high-dose chemotherapy with autologous stem cell transplant (ASCT). 

The inflammatory cascade induced by irradiation can be mitigated in rodent models by non-steroidal anti-inflammatory drugs (pioglitazone, fenofibrate, angiotensin type 1 receptor antagonists) [52] or bone marrow-derived mesenchymal stem cells [53] or by voluntary aerobic exercise [54,55].

Several neuroprotective agents, such as antioxidants, flavonoids, valproic acid, fingolimod, ramipril, are being investigated in the prevention/treatment of cognitive defects from radiation [56].

The deuteration of drinking water has been suggested to protect mice from whole-body gamma irradiation [57].

Hyperbaric oxygen therapy has been suggested as a treatment for late radiation injuries to tissues of head and neck, but thus far there is no evidence of benefit in clinical outcome with established radiation injury to neural tissue [58].

## 4. Radiation-Induced Late Cerebrovascular Complications

Radiation-induced arteriopathy may be an important cause of stroke in patients after radiotherapy. 

Focal arteriopathies and Moyamoya arteriopathy have been described post radiation, with an estimated cumulative incidence of up to 12% at 10 years among children who survived brain tumors [59,60]. Moyamoya syndrome is a potentially late severe complication in children after cranial irradiation, especially for tumors close to the circle of Willis, such as optic pathway glioma, and those patients who receive higher doses (>50 Gy) [60]. Patients with brain tumors who received radiation to central areas of the brain are also more vulnerable to cerebrovascular mortality [61]. 

Intracerebral microbleeds generally occur several years post radiation, with a cumulative incidence ranging from 40% to 90% by 5–10 years after radiation, and susceptibility-weighted imaging is superior to T2*-weighted gradient echo [62,63]. 

Delayed radiation injury may result in proliferative vascular lesions, such as capillary telangiectasia and cavernoma, when using radiation doses of >30 Gy, which can lead to intracranial hemorrhage and cause devastating neurological sequelae [64]. The reported latency period for radiation-induced cavernomas ranges from 1–26 years [64] and 3–9 months for telangiectasias [65].

## 5. Brain Injury Following Particle Therapy

### 5.1. Clinical Aspects

An increasing number of studies has suggested a reduction in late adverse effects with protons in comparison with photons of standard radiotherapy, including neurocognitive sequelae [66,67], endocrine abnormalities [68], hearing and visual disturbances [69], and secondary malignancies [70]. Proton beam therapy (PBT) offers dosimetric advantages over conventional photon-based radiotherapy (XRT): the absence of an exit dose due to the so-called “Bragg peak phenomenon” may allow a better sparing of adjacent distal normal brain critical structures [71], such as optic pathways, brain stem, pituitary, and auditory structures. For patients needing craniospinal irradiation, proton beams used to treat the spine cease before exposing adjacent organs at risk, thus eliminating the risk of radiation-induced heart disease and secondary tumors. A clear example of ideal targets for PBT are radioresistant skull base tumors (chordomas, chondrosarcomas), that require radiation doses exceeding the tolerance doses of the surrounding critical structures. In pediatric tumor types, with a large proportion of long-term survivors (pilocytic astrocytomas, medulloblastomas, ependymomas), there is a strong rationale for using PBT to minimize late toxicities. However, most of these tumor types are located in the posterior fossa, and the potential brain stem toxicity following PBT has become an issue [72]. Symptomatic brain stem injury (SBI), including pathologically verified radiation necrosis or newly appeared areas of contrast enhancement on MRI following PBT, has been reported to be either 3.8% at 2 years [73] or 2% at 5 years [74] or 3.2% as crude incidence [75]. These values are similar to those reported with standard XRT [76]. Younger age, higher radiation doses, and pre-treatment with high-dose chemotherapy are considered risk factors for SBI after PBT. The frequency of transitory MRI changes following PBT for ependymomas or low-grade gliomas has been reported to be either higher [77,78] or equal to [79] that following conventional XRT. A high proportion of transitory MRI changes has been reported also after carbon ion radiotherapy of head and neck and skull base tumors [80]. Overall, a major limitation is that most studies are observational and, even if comparative, the nature is retrospective. 

The risk of radionecrosis following protons seems similar to that of photons, while there are few data so far following carbon ions.

Radiation-induced microbleeds have been reported in children after proton irradiation [81].

Proton stereotactic radiosurgery has been used in the last years to treat brain metastases, and CNS toxicities seem similar to those reported with photon stereotactic radiosurgery [82] (Table 2).

### 5.2. Pathophysiology

Animal models suggest that the negative effects of protons on cognition are qualitatively similar to that of photons, even at low doses [83]. 

Similar to photon exposure, proton exposure induced in the dentate gyrus and SGZ of the hippocampus and a reduction in neurogenesis due to the high sensitivity of neural precursor cells to radiation [84,85]. Moreover, low-dose (1 Gy) proton exposure in mice resulted in persistent changes in the number, density, and structure of dendritic spines [86]. Proton therapy may negatively impact the synaptic transmission, with an enhanced release of the inhibitory neurotransmitter GABA (gamma-aminobutyric acid) [87].

Microglial activation following protons has been observed [88].

Recently, a preclinical model of late side effects from proton irradiation has been developed [89]. The authors have investigated the dose- and time-dependent responses of mouse brain to the irradiation of hippocampal regions, with follow-up examinations for up to 6 months. MRI contrast agent leakage (due to the damage to the BBB) occurred in the irradiated brain areas, and were earlier and progressive in the high-dose groups. Mouse health status and survival paralleled the extent of contrast enhancement on MRI. Histologic analysis showed vessel damage, gliosis, and granule cell dispersion, which partly involved the non-irradiated contralateral hippocampus in the higher dose groups. However, some experimental studies did not find negative effects of protons on cognition [90,91].

### 5.3. Strategies to Minimize Brain Damage Following Particle Therapy

In order to minimize normal brain toxicity following particle therapy, studies have tried to correlate biological outcomes to physical factors. A lower risk of SBI in pediatric brain tumors has been observed after adopting strict brain stem dose constraints [75]. Other than dose, the patterns of energy deposition differ depending on ion type (i.e., proton vs carbon ions) and energy: thus, factors such as LET (Linear Energy Transfer) should be incorporated into the treatment planning process [72,92,93]. Thus far, hippocampal avoidance proton therapy has not been investigated in order to minimize the damage to neural precursors. The same is true for the potential role of anti-inflammatory drugs or physical exercise.

## 6. The Issue of Radionecrosis Following Stereotactic Radiosurgery 

Stereotactic radiosurgery (SRS), either in single or multiple fractions, has for several years been considered since the gold standard for the treatment of brain metastases [94,95]. A typical feature of brain damage following SRS is radionecrosis, for which the definition in the literature is based either on tissue biopsy showing necrosis, vessel damage, inflammation with absent or minimal tumor persistence or on MRI only displaying increase of contrast enhancement, necrosis, edema, and mass effect. When changes tend to reduce over time, the term pseudoprogression is used. Radionecrosis/pseudoprogression is characterized by an increase in focal neurological symptoms, including headache and seizures, with a latency from treatment that is highly variable (from few months to more than 2 years), and is difficult to distinguish from true tumor progression on conventional neuroimaging.

Advanced imaging techniques can improve the differential diagnostic capabilities [96,97]. Diffusion-weighted imaging (DWI) may show, in cases of radiation necrosis, an elevated apparent diffusion coefficient (ADC), while in case of recurrent tumor, ADC is commonly lower. Perfusion imaging with the calculation of relative cerebral blood volume (rCBV) seems to be the most useful technique: rCBV is often elevated in case of a recurrent tumor with high angiogenesis (more in glioblastoma than in brain metastasis), while it is low in radiation necrosis with minimal persistent tumor. Conversely, MR spectroscopy is of limited value. 

PET imaging may be a useful tool if there is still uncertainty on MRI [98]. As an 18F-FDG tracer is of limited usefulness due to the high background uptake by normal brain in the cortex and basal ganglia, amino acid radiotracers (11C-MET, 18F-FET,18F-DOPA) are now increasingly utilized [99,100]. 18F-fluciclovine, a new amino acid tracer for PET imaging, has been recently suggested to be of increased usefulness for differentiation radionecrosis from tumor recurrence after the treatment of brain metastasis with stereotactic radiosurgery [101]. Several radiomic signatures are under investigation and have been preliminarily suggested to be of potential utility [102,103]. 

Overall, the incidence of radionecrosis following SRS for brain metastases is reported in up to 30% of patients across studies [104,105].

Some factors are commonly associated with an increased risk of radiation necrosis, including treatment volume, dose fractionation schedule, prior radiotherapy, association with chemotherapy, tumor site, and histologic type [106].

More recently, the risk of radionecrosis following SRS to the resection cavity has gained attention. The values range between 9% and 17.5% [107,108]. The actuarial risk could increase over time: 7% at 1 year up to 16% at 2 years [109], and a steroid dependency is not infrequent. Preoperative SRS could reduce the risk of radiation necrosis [110]. 

There is evidence supporting an increased risk of radionecrosis following SRS in combination with immuno-checkpoint inhibitors (in particular, ipilimumab) for brain metastases from melanoma [111,112].

The risk of radionecrosis seems to be higher in brain metastases from breast cancer with the concurrent administration of SRS with HER2-directed therapies [113], in particular, TDM1 [114,115]. 

The risk of radionecrosis following the treatment with BRAF inhibitors in melanoma is still debated [116].

Radionecrosis is commonly treated with steroids. The antivascular endothelial growth factor agent bevacizumab may allow the stabilization/normalization of the vascular permeability in patients unresponsive to steroids [117,118,119]. Laser interstitial thermal therapy or surgical resection can be useful in some patients [120] (Table 3).

## 7. New Approaches to Investigate Neurotoxicity from Treatments

Conventional MRI has demonstrated a reduction in hippocampal volume following high-dose conformal radiotherapy in patients with primary brain tumors [121]. Likewise, a dose-dependent reduction in cortical thickness, especially in temporal and limbic lobes, 1 year after partial brain irradiation of high-grade gliomas has been observed [122]. In addition to hyperintensity in T2 or FLAIR images, seen with anatomical MRI, the damage to the white matter from radiation is better depicted with DTI techniques, which may show microstructural changes. Fiber tracking procedures, based on advanced DWI methods, have shown after multimodal therapy of gliomas a significant reduction in local white matter fiber density [123]. Compared to a matched cohort of healthy subjects, the reduction was strong in contrast enhancing lesions and in regions with increased FET PET uptake, and still pronounced in regions with T2-FLAIR hyperintensity. Interestingly, the total fiber loss was associated with significantly lowered performance status. It remains to be elucidated whether this loss of fiber density on MRI tractography is correlated with neuronal dysfunction in transmission of action potentials along the axons in the affected areas.

Functional MRI, which indirectly measures neural activity through the measurement of changes in blood flow, has observed that after high radiotherapy doses there is a decrease in neural activation during motor and sensory tasks that lasts several months [124]. Moreover, MR spectroscopy has reported a molecular derangement not only in areas of the brain receiving high doses of radiotherapy, but also in unexposed regions [125,126], probably related to a release of cytokine cascades.

In addition to advances in neuroimaging, liquid biopsies of blood and CSF are now an emerging tool of research to identify early indicators of CNS injury following therapies (radiotherapy and/or chemotherapy).

Recently, extracellular vesicles in the serum of mice receiving radiotherapy have been shown to carry increased levels of GFAP (an astrocyte marker) and protein-bound 4-hydroxy-2-nonenal (HNE) (an oxidative damage marker) [127]. A secondary analysis of a phase III trial on brain metastases has reported the role of some circulating biomarkers in predicting a higher risk of cognitive failure after WBRT [128].

Primary CNS lymphomas, who typically undergo aggressive multimodality treatments (high-dose methotrexate, high-dose ARA-C, WBRT) now represent a field of investigation regarding brain damage from treatments. A recent study has reported that elevated CSF lactate, associated with lower gamma-aminobutyric acid (GABA) and higher glutamate/GABA ratio were strongly correlated with decreased cognitive functions (as measure by MMSE scores) [129].

A higher level of CSF tau protein (a marker of neuronal damage) has been correlated with diminished hippocampal cell proliferation in mice exposed to high-dose methotrexate [130].

Overall, there is now a rationale for further exploring in well-designed clinical studies the correlations of biotumoral markers of neurodegeneration and neurocognitive functions in patients, such as PCNSL or brain metastases, who undergo aggressive treatments.

## 8. Conclusions

A major factor that has limited the progress in the knowledge of mechanisms of damage of the brain from radiotherapy in the human setting is the lack of animal models that mimic the clinical situations. As a matter of fact, most of the animal models have used radiation modalities that are far from those employed in the clinic (whole-body radiation therapy, single high-dose fractions, absence of brain tumor).

Thus far, there is not an optimal strategy to prevent or minimize the risk of brain damage from radiation in humans. In clinical practice, whole-brain radiotherapy with hippocampal sparing is largely utilized in US and Europe, but the advantage in terms of long-term protection is unknown. It must be said that the extensive use of radiosurgery is reducing the use of WBRT. Conversely, the role of commercially available drugs, such as memantine, donepezil, or antidepressants, is simply palliative. There is need in the future for well-designed clinical trials on the new compounds or techniques that will emerge from the new compounds or techniques that will emerge from basic research. In this regard, the choice of measurable endpoints and controls will be a critical issue. 

In contrast, the continuous advances in molecular biology, advanced neuroimaging [131,132], and liquid biopsy will allow the acquisition of new critical information on early biomarkers of brain tissue damage and/or predictors of long-term cognitive outcome.

## Figures and Tables

**Table 1:** Neurotoxicity from WBRT: Key Points.

Neurotoxicity from WBRT: Key Points
Cognitive decline may be a consequence of whole-brain radiotherapy (WBRT) in brain metastases and primary CNS lymphomas.
The pathogenesis of cognitive decline following WBRT is multifactorial, including reduced neurogenesis, neuro-inflammation, functional damage to neurons, and disruption of the blood–brain barrier.
Cognitive decline following WBRT may be mitigated either by hippocampal avoidance or lowering the total radiation dose or use of drugs with neuroprotective or anti-inflammatory potential.

**Table 2:** Neurotoxicity from Proton Therapy: Key Points.

Neurotoxicity from Proton Therapy: Key Points
Symptomatic brain stem injury (SBI) may be a consequence of proton irradiation of tumors in the posterior fossa, especially in children.
Radiation necrosis and transitory newly appeared areas of contrast enhancement on MRI are the two major forms of SBI.
The adverse effects of proton therapy may be minimized by strict brain stem dose constraints.

**Table 3:** Radionecrosis Following Stereotactic Radiosurgery: Key Points.

Radionecrosis Following Stereotactic Radiosurgery: Key Points
Radionecrosis/pseudoprogression following stereotactic radiosurgery is a major issue in the treatment of brain metastases.
The incidence is up to 30% of patients, and factors associated with an increased risk are treatment volume, dose fractionation schedule, prior radiotherapy, association with immune checkpoint inhibitors in brain metastases from melanoma and within HER-2 directed therapies in brain metastases from breast cancer.
As radiation necrosis is a consequence of a focal damage of the blood–brain barrier, corticosteroids, and the anti-VEGF compound bevacizumab are the major players in the medical treatment.

## Data Availability

No new data were created or analyzed in this study. Data sharing is not applicable to this article.
